# Supplementary material for: Are diffusion models ready for materials discovery in unexplored chemical space?
Source: Patterns (N Y). 2026 Apr 13;7(6):101537. doi: 10.1016/j.patter.2026.101537 (PMC13280719; doi:10.1016/j.patter.2026.101537)
Supplement: Document S1. Figures S1 and S2, Tables S1 and S2, and supplemental note [file mmc1.pdf]

Patterns, Volume *7*

Supplemental information

Are diffusion models ready for materials discovery in unexplored chemical space?

Sanghyun Kim, Gihyeon Jeon, Seungwoo Hwang, Jiho Lee, Jisu Jung, Seungwu Han, and Sungwoo Kang

Supplemental Note

Proof of Size-Dependence of KL Divergence in Diffusion Models.

To demonstrate that the discrepancy between the noise and stable structure scales with the number of atoms N, we calculate the Kullback-Leibler divergence $D_{\mathrm{KL}}(p(\mathbf{x}_0)||p(\mathbf{x}_T))$. By the gradient theorem, we have

$$\log p(\mathbf{x}_0) - \log p(\mathbf{x}_T) = \int_{\text{path}} \nabla \log p(\mathbf{x}_t) \cdot \mathrm{d}\mathbf{x}_t = \int_T^0 \nabla \log p(\mathbf{x}_t) \cdot \frac{\mathrm{d}\mathbf{x}_t}{\mathrm{d}t} \mathrm{d}t.$$

For the variance exploding stochastic differential equation[S1] (VE-SDE), deterministic process corresponding to forward diffusion is given by

$$\frac{\mathrm{d}\mathbf{x}_t}{\mathrm{d}t} = -\frac{1}{2} g(t)^2 \nabla \log p(\mathbf{x}_t).$$

Thus we have

$$\int_T^0 \nabla \log p(\mathbf{x}_t) \cdot \frac{\mathrm{d}\mathbf{x}_t}{\mathrm{d}t} \mathrm{d}t = \int_T^0 \nabla \log p(\mathbf{x}_t) \cdot \left(-\frac{1}{2} g(t)^2 \nabla \log p(\mathbf{x}_t)\right) dt$$
$$= \frac{1}{2} \int_0^T g(t)^2 ||\nabla \log p(\mathbf{x}_t)||^2 \mathrm{d}t.$$

Hence, Kullback-Leibler divergence is given by

$$D_{\mathrm{KL}}\big(p(\mathbf{x}_0)||p(\mathbf{x}_T)\big) = \frac{1}{2} \int_0^T g(t)^2 \mathbb{E}[||\nabla \log p(\mathbf{x}_t)||^2] \mathrm{d}t.$$

By applying Tweedie's formula[S2], we have $\nabla \log p(\mathbf{x}_t) = \frac{1}{\sigma_t^2}(\mathbb{E}[\mathbf{x}_0|\,\mathbf{x}_t] - \mathbf{x}_t)$ and

$$\mathbb{E}[||\nabla \log p(\mathbf{x}_t)\,||^2] = \frac{1}{\sigma_t^4} \mathbb{E}[||\mathbb{E}[\mathbf{x}_0|\mathbf{x}_t] - \mathbf{x}_t||^2] \quad (1)$$
$$= \frac{1}{\sigma_t^4} \mathbb{E}[||\mathbf{x}_t||^2 - 2\mathbf{x}_t \cdot \mathbb{E}[\mathbf{x}_0|\,\mathbf{x}_t] + ||\mathbb{E}[\mathbf{x}_0|\,\mathbf{x}_t]||^2]$$
$$= \frac{1}{\sigma_t^4} \big(\mathbb{E}[||\mathbf{x}_t||^2] - 2\mathbb{E}\big[\mathbf{x}_t \cdot \mathbb{E}[\mathbf{x}_0|\,\mathbf{x}_t]\big] + \mathbb{E}[||\mathbb{E}[\mathbf{x}_0|\,\mathbf{x}_t]||^2]\big).$$

The first term is given by:

$$\mathbb{E}[||\mathbf{x}_t||^2] = \mathbb{E}[||\mathbf{x}_0 + \sigma_t \mathbf{z}||^2]$$
$$= \mathbb{E}[||\mathbf{x}_0||^2] + \sigma_t^2 \mathbb{E}[||\mathbf{z}||^2] + 2\sigma_t \mathbb{E}[\mathbf{x}_0 \cdot \mathbf{z}]$$
$$= \mathbb{E}[||\mathbf{x}_0||^2] + \sigma_t^2 3N + 0$$

where $\mathbf{z} \sim N(\mathbf{0}, \boldsymbol{I})$ is a $3N$-dimensional standard Gaussian noise vector. The second term is derived as follows:

$$\begin{aligned}\mathbb{E}\big[\mathbf{x}_t \cdot \mathbb{E}[\mathbf{x}_0 | \, \mathbf{x}_t]\big] &= \mathbb{E}[\mathbf{x}_t \cdot \mathbf{x}_0] \\ &= \mathbb{E}[(\mathbf{x}_0 + \sigma_t \mathbf{z}) \cdot \mathbf{x}_0] \\ &= \mathbb{E}[\|\mathbf{x}_0\|^2] + \sigma_t \mathbb{E}[\mathbf{z} \cdot \mathbf{x}_0] \\ &= \mathbb{E}[\|\mathbf{x}_0\|^2] + 0.\end{aligned}$$

The third term is obtained using the orthogonality principle,

$$\mathbb{E}[\|\mathbb{E}[\mathbf{x}_0 | \, \mathbf{x}_t]\|^2] = \mathbb{E}[\|\mathbf{x}_0\|^2] - \mathbb{E}[\|\mathbf{x}_0 - \mathbb{E}[\mathbf{x}_0 | \, \mathbf{x}_t]\|^2]$$

where the last term represents the minimum mean squared error (MMSE), as $\mathbb{E}[\mathbf{x}_0 | \, \mathbf{x}_t]$ is the posterior mean. Therefore equation (1) is given by

$$\begin{aligned}\mathbb{E}[\|\nabla \log p(\mathbf{x}_t)\,\|^2] &= \frac{1}{\sigma_t^4}\left(\mathbb{E}[\|\mathbf{x}_0\|^2] + \sigma_t^2 3N - 2\mathbb{E}[\|\mathbf{x}_0\|^2] + \mathbb{E}[\|\mathbf{x}_0\|^2] - \mathrm{MMSE}\right) \\ &= \frac{1}{\sigma_t^4}\left(\sigma_t^2 3N - \mathrm{MMSE}\right) \\ &= \frac{3N}{\sigma_t^2} - \frac{\mathrm{MMSE}}{\sigma_t^4}.\end{aligned}$$

Given that $g(t)^2 = \frac{\mathrm{d}\sigma_t^2}{\mathrm{d}t}$ in the VE-SDE, Kullback-Leibler divergence is calculated as follows.

$$\begin{aligned}D_{\mathrm{KL}}\big(p(\mathbf{x}_0)\|p(\mathbf{x}_T)\big) &= \frac{1}{2}\int_0^T \frac{\mathrm{d}\sigma_t^2}{\mathrm{d}t}\left(\frac{3N}{\sigma_t^2} - \frac{\mathrm{MMSE(t)}}{\sigma_t^4}\right)\mathrm{d}t \\ &= \frac{3N}{2}\int_0^T \frac{1}{\sigma_t^2}\frac{\mathrm{d}\sigma_t^2}{\mathrm{d}t}\mathrm{d}t - \frac{1}{2}\int_0^T \frac{\mathrm{MMSE}(t)}{\sigma_t^4}\frac{\mathrm{d}\sigma_t^2}{\mathrm{d}t}\mathrm{d}t \\ &= 3N \log\frac{\sigma_T}{\sigma_0} - \frac{1}{2}\int_0^T \frac{\mathrm{MMSE}(t)}{\sigma_t^4}\frac{\mathrm{d}\sigma_t^2}{\mathrm{d}t}\mathrm{d}t.\end{aligned}$$

Consequently, the first term explicitly demonstrates the linear dependence on the number of atoms, showing that $D_{\mathrm{KL}}\big(p(\mathbf{x}_0)\|p(\mathbf{x}_T)\big) \propto 3N \log\frac{\sigma_T}{\sigma_0}$.

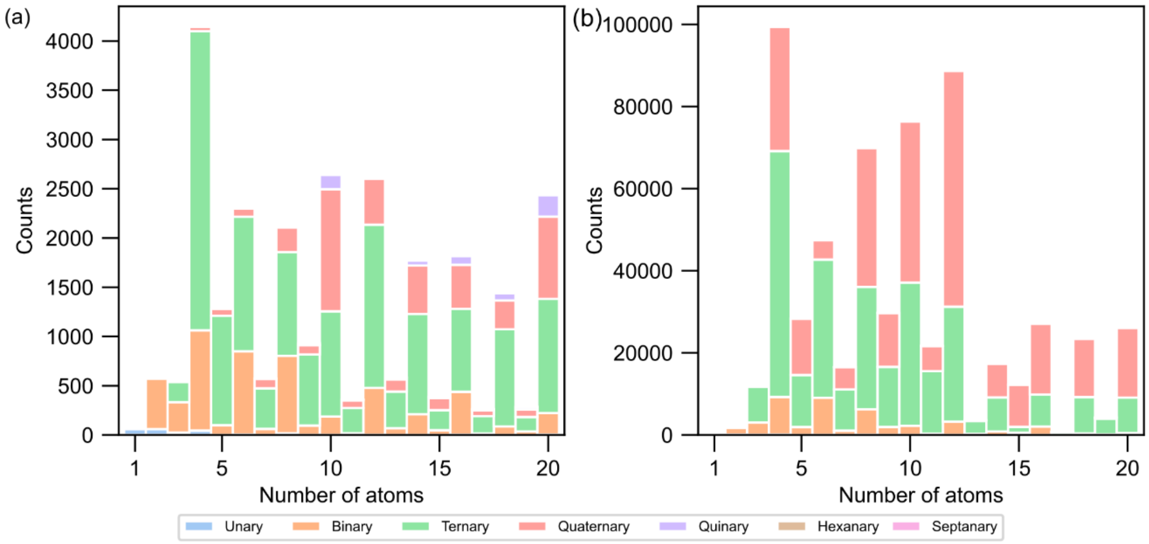

Figure S1. Element number distribution of (a) MP-20 and (b) Alex-MP-20 datasets.

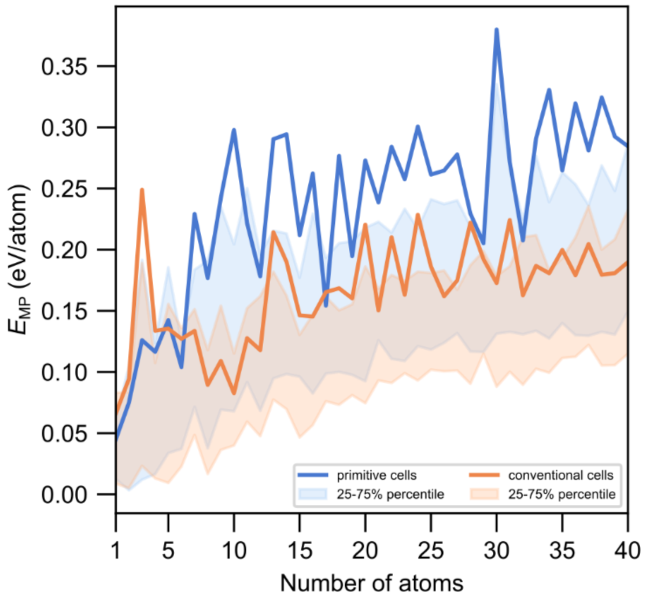

Figure S2. E_{MP} as a function of number of atoms for the MatterGen models trained with primitive and conventional cells.

Table S1. AFLOW-XtalFinder results for GNoME test materials.

Formula	Prototype
Ir5Mo2Ru1	Mo1Ir3
Be8Co1V3	Be2V1
Ag4O16Te4	Mg1Te1O4
Nb1Te8Zr5	(Ti,Nb,V)3(S,Se,Te)4
Ge1Lu12Tc3	Fe3C
Pd1Rh2Se6	Te2Ir
Cu1Sn4Tc1	Al2Cu
Er20Ir4Si12	Er5Si4
As2F14Ni2	CuAsF7
K1O8Ta5	x
La4Li4S8	LaLiO2
O18Ta4Yb8	Mg4Ta2O9
CrGa3O6	Ga2O3
CsDy3S5	CsEr3Se5
La2PtI2	I2La2As
LuH2Br	BrH2Tb
Tb4OsCl4	Er4OsBr4
Tm5GeSe4	TmSe
UBr5Cl	MoCl6
Y3Cl5	La3Cl5
Al4Ga4N4V12	Ga2N1V3
Ir8N4Si4Zr12	Ti3Al2NiN
Ag1I1La10Pb6	La5AgPb3
Dy2F2O12Sb4	Nd1Ta2O6F1
Br3Hg6Li3O6	Na1Hg2I1O2
C2Dy2I2Pr2	Pr2C1Br1
Br18C4Dy10Rb1	Cs1Er10C4I18
Ce1N2Np1Sb1	Th2SbN2
F24Li2Na6Pd4	Na3Li1Ti2F12
Ba2Li2O6Y2	CaRhO3
Ag2As1Na1S4	Ag2FeSnS4
As4Co4O16Rb4	RbZnPO4
Cu4K4Mn2S8	Cu2Rb2TiS4
Gd4In6O18Pr2	YInO3
Li1O8Tc3Yb1	LiInMo3O8
Dy5O12Re1Ru1	Dy5Mo2O12
Li12Nb3O16Ta1	Li3NbO4
BaLiTbSe3	CsTbMnSe3
RbLi4NbO5	KLi4FeO5
TbHoP2S2	ErPS
Ge2I2Ir2Pr4	HPC-Bi2O3
Gd3Ir4Na1O14	x
B1Rh2Ru4Ta2	δ-Bi2O3
Br1Cl3Te4Th2	x
Cd1S12Sn1Ta6	x

Er1I10Ni1Tm5	x
In4Lu13Rh4Tm3	Gd4RhIn
Bi2Ca4Pb2Pt4	MnCuP
La4O20Pr2U4	CaSi2
Er3Hg2In3Ir1	GdSI
Hf8N4Rh8Zr8	x
Al6Ca1Cu4Pr1	CoSn
Br10Co1La2Tb4	x
As1Dy4Ge7Rh16	Ni4B3
Er5Ir6Sn6Zr1	ZrNiAl
Au2Co4Te8	Al2MgO4
Sc1Si16V12Zr7	x
Mn2N6Na8	x
Br20Cd2Er4	x
Sc1Si5Sn1Tb9	Mn5Si3
Cs8Mg2P4S16	x
K1P6Pd21Tl1	x
Fe5I16Pb1	x
C12Cr3La6Mo1	x
Cs8Mo1Se16W3	K2SO4
Bi1Co4Cu1Tm12	x
Er1Ho1Nd8Pb6	Mn5Si3
Ga4Pd7Pr4Pt1	Legacy prototype
Al1Fe1Os2Tb2	x
Cs8Mo6N16Zn2	Legacy prototype
Al2Au2In1Pd1Th3	x
Ac6Bi1Cd18Mg5	x

Table S2. Training loss for models trained under different settings: inclusion or exclusion of cosine similarity in the score function, training mode (chemical-formula-fixed (CSP) or chemical-formula-free), and training data type (primitive or conventional).

Total loss (0.1A + B + C)	Position loss (A)	Cell loss (B)	Atom-type loss (C)	Cosine similarity	Data lattice
0.376	2.45	0.0225	0.109	w/	primitive
0.379	2.43	0.0228	0.113	w/	conventional
0.868	2.86	0.4622	0.117	w/o	primitive
0.853	2.77	0.4624	0.113	w/o	conventional
0.226	2.09	0.0166	CSP mode	w/	primitive
0.234	2.15	0.0187	CSP mode	w/	conventional
0.676	2.41	0.4350	CSP mode	w/o	primitive
0.669	2.41	0.4290	CSP mode	w/o	conventional

References

[S1] Song et al., Score-based generative modeling through stochastic differential equations. **2020**, arXiv:2011.13456.
[S2] Bradley Efron, Tweedie's formula and selection bias. **2011**, Journal of the American Statistical Association, 106.496: 1602-1614.
